# Field electron emission properties of bulk diamond/expanded graphite composite cathode

**DOI:** 10.1016/j.isci.2025.114043

**Published:** 2025-11-14

**Authors:** Qianyu Ji, Yihui Zhang, Jiacheng Zhang, Wenhua Guo, Jiyuan Zhao

**Affiliations:** 1 School of Mechanical Engineering, Xi’an Jiaotong University, Xi’an, Shaanxi 710049, P.R. China; 2 The State Key Laboratory for Manufacturing Systems Engineering, Xi’an Jiaotong University, Xi’an 710054, Shaanxi, P.R. China; 3 National Innovation Institute of Additive Manufacturing, #997, Shanglinyuan 8th Road, Hi-tech District, Xi’an 710054, Shaanxi, P.R. China; 4 School of Automation, Beijing Information Science and Technology University, Beijing 100192, P.R. China

**Keywords:** Electrochemistry, Energy materials

## Abstract

Diamond exhibits a negative electron affinity potential and a high thermal conductivity. Nevertheless, the elevated surface resistivity of diamond restricts its utilization in cold cathodes. Expanded graphite, an allotrope of diamond, is a material that exhibits high electrical conductivity and is cost-effective to manufacture. The combination of these two elements into a hybrid structure is a highly desirable proposition, as it would facilitate a synergistic approach to field-emitting materials. In this study, diamond-expanded graphite bulk composite cathodes were prepared using the cold pressing method. The results demonstrate that the cathode comprising diamond particles with a diameter of 0.5 μm and a content of 20% exhibits the most favorable electron emission activity. The turn-on field strength (at 1 mA/cm^2^) and threshold field strength (at 10 mA/cm^2^) were 1.61 V/μm and 2.11 V/μm, respectively. The maximum current density and the field enhancement factor reached 911.24 mA/cm^2^ (at 3.77 V/μm) and 4490, respectively.

## Highlights

• Diamond and expanded graphite composite cathodes have enhanced field emission performance
• Adjusting diamond size and content modulates field emission performance and lifetime
• Heat treatment can dramatically improve field emission stability

## Graphical abstract

**Figure:**
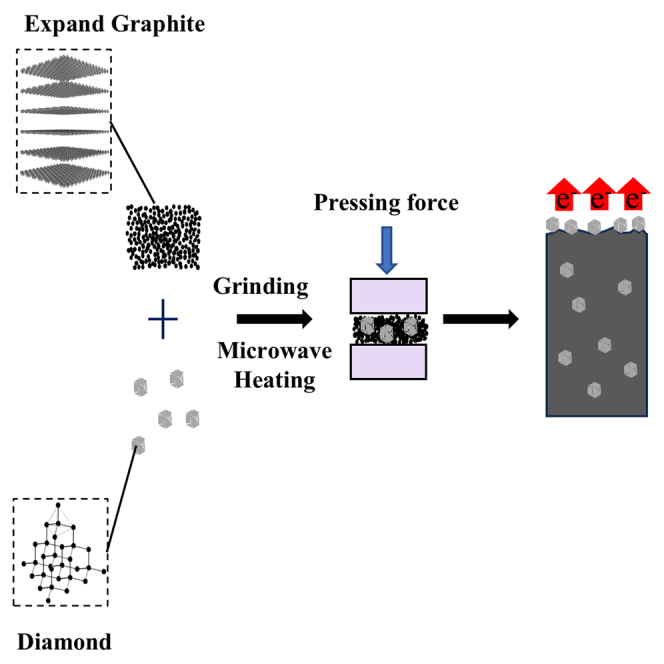

## Introduction

In the domain of electron field emission, X-rays and extreme ultraviolet (EUV) rays, carbon-based materials have become the most prevalent and widely recognized materials. This is due to several factors, including their unique structure and dimensions, low and stable electron work function values, high mechanical robustness, and electrical and thermal conductivity.^1^^,^^2^^,^^3^^,^^4^^,^^5^ Among the most well-known major isomers of carbon-based materials are graphite and diamond, which exhibit completely distinct structural and electrical properties. Graphite is distinguished by an sp^2^ hybridized and layered van der Waals bond structure, exemplary lubricating properties, and high electrical conductivity, yet it is mechanically soft.^6^^,^^7^ Diamond exhibits an sp^3^ carbon in-wafer and tetrahedral structure, demonstrating elevated levels of hardness, a high Young’s modulus, a broad band gap, and a low work function. Additionally, it exhibits high thermal conductivity and a low electron affinity. Nevertheless, it functions as an electrical insulator.^8^ Despite the excellent properties exhibited by these materials, they are subject to limitations when it comes to field emission alone. For instance, diamond demonstrates substandard electrical conductivity, leading to a minimal emission current density (117 μA/cm2 at 20 V/μm).^9^

In order to circumvent these shortcomings and exploit the superior characteristics of carbon-based materials to their fullest extent, several composite/hybrid cathode structures have been developed.^10^^,^^11^ In a previous study, Santos et al. employed microwave plasma chemical vapor deposition (MPCVD) to synthesize graphene-diamond hybrids on copper substrates. The turn-on field strengths were found to range from approximately 4.6 to 8.4 V/μm and decrease with increasing nanodiamond cluster content.^12^ Huang et al. enhanced the field emission efficiency and stability by modifying carbon nanowalls with ultrananocrystalline diamond (UNCD) particles via an electrostatic self-assembly seeding process, followed by a brief period of growth in plasma-chemical vapor deposition (PCVD). The turn-on field strength was reduced from 3.0 V/μm for bare carbon nanowalls to 1.8 V/μm for diamond modification, while the current density was increased by approximately an order of magnitude.^13^ In a study conducted by Li et al., the deposition of diamond-graphite nanohybrid films on Si substrates using MPCVD resulted in the enhancement of electron field emission. The emission current density of the diamond-graphite nanohybrid film was found to be eight times higher than that of the UNCD film under the same applied electric field.^14^ Furthermore, the fabrication of composite/hybrid materials enables substantial enhancements in a variety of properties, including electrical, mechanical, chemical, optical, and thermal properties.^15^^,^^16^

Expanded graphite, a carbon-based material with a loose and porous nature, has been employed in the preparation of a diverse range of conductive composites for various applications, including hydrogen storage,^17^ thermal storage,^18^ fuel cells,^19^ batteries,^17^ supercapacitors,^20^ and sensors.^21^ The substance is utilized due to its exceptional chemical and thermal stability, high electrical conductivity, and cost-effectiveness, which render it an invaluable asset in a multitude of applications. Nevertheless, there has been comparatively little research conducted on expanded graphite composites in the field of electron emission. Most studies have concentrated on determining the threshold conditions for field emission and achieving high-current emission.^22^^,^^23^^,^^24^^,^^25^ Furthermore, there is a dearth of research examining the influence of diamonds on field emission characteristics and the durability of field emission.

In accordance with the methodology employed in this study, the diamond and expanded graphite (D-EG) particles were mixed, and bulk composite cathodes were prepared by the cold pressing method. Subsequently, the impact of diamond content and particle size on field emission characteristics was subjected to a comprehensive investigation.

## Results and discussion

### Structural and morphological analysis

**Figure 1:**
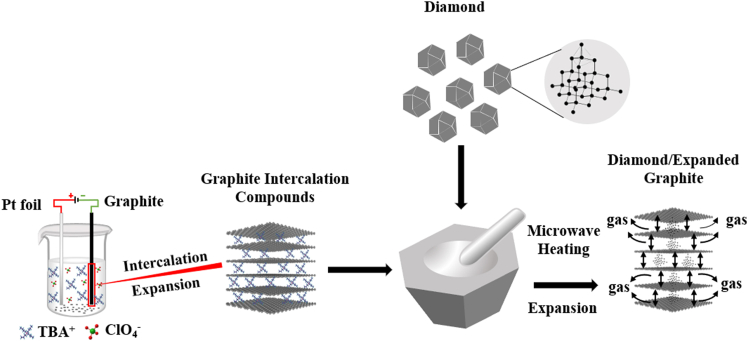
The following schematic illustration depicts the synthetic route employed for the production of diamond and expanded graphite composite compounds

Figure 1 shows the synthesis route for the diamond-expanded graphite composite. Table 1 lists the average thickness of the final composite bulk cathodes for different diamond sizes and contents.

**Table 1:** Diamond granule size, content distribution, and thickness of cathodes C1–C6

Cathode	Diamond granule size (μm)	Content (%)	Thickness (μm)
C_1_	–	0	384.7
C_2_	1.15	30	281.9
C_3_	1.15	20	293.7
C_4_	1.15	10	371.2
C_5_	0.50	20	365.8
C_6_	0.25	20	296.6

**Figure 2:**
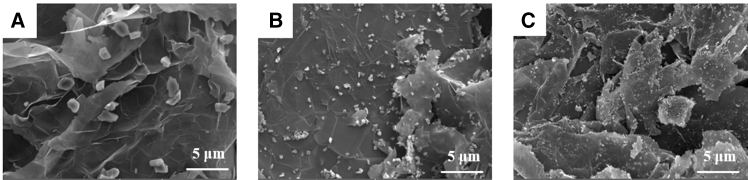
Material characterization of composite cathodes (A–C) SEM images of D-EG powders with 20% diamond and granule sizes of (A) 1.15 μm, (B) 0.5 μm, and (C) 0.25 μm, respectively.

Figure 2 presents the SEM images of D-EG powders with 20% diamond content and sizes of 1.15μm, 0.5 μm, and 0.25μm, respectively. It can be observed that all diamond particles adhere uniformly to the surface of EG.

**Figure 3:**
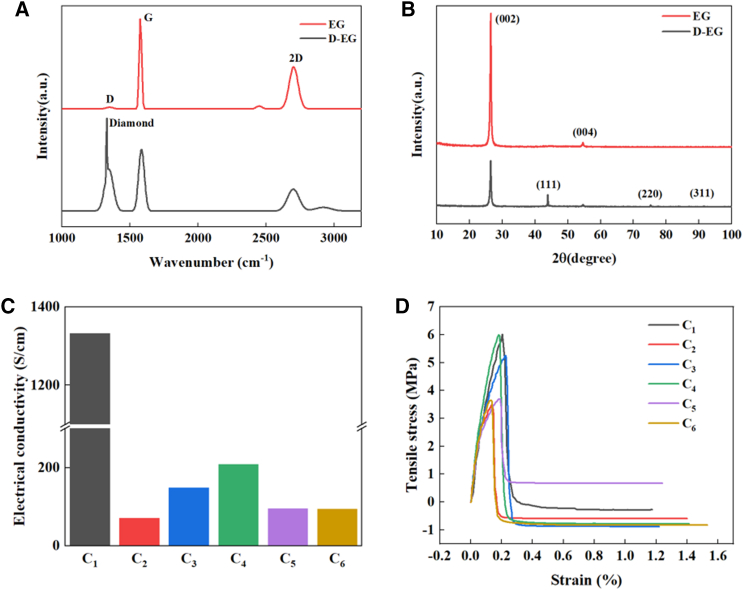
Structural and physical properties analysis of composite cathodes (A–D) (A) Raman spectra and (B) XRD patterns of EG and D-EG, (C) electrical conductivity, and (D) mechanical properties of bulk cathodes.

To determine whether GICs has fully expanded and the degree of defect in the EG and D-EG, Raman spectroscopy, and XRD measurements were performed. Figure 3A illustrates the Raman spectra of EG and D-EG powders within the range of 1000–3200 cm^−1^. The peak observed at approximately 1329 cm^−1^ is attributed to the stretching vibration of the tetrahedral (sp^3^) bonded diamond lattice, which is a defining characteristic of the diamond structure.^26^ The D peak is located at about 1349 cm^−1^ and is associated with defects in sp^2^ graphite flakes, polycrystalline graphite, and graphite-like materials with crystalline defects; the G peak occurs at 1580 cm^−1^, which is due to the bond stretching of all the sp^2^ hybridized pairs of atoms; and the 2-fold frequency peak of the D peak (2D peak) occurs at about 2690 cm-1, which splits into 2 components, 2D_1_ and 2D_2_, in polycrystalline graphite samples.^27^ The ratio of I_D_/I_G_ is commonly used to characterize the degree of defects in graphitic materials. The calculated I_D_/I_G_ for EG and D-EG are 0.019 and 0.609, respectively, indicating that the degree of defects in EG becomes larger after the addition of diamond particles and grinding.

The X-ray diffractograms of EG and D-EG powders are shown in Figure 3B. After the microwave expansion of GICs, peaks located at 9.4°, 19.2°, 26.57°, and 27.97° disappeared,^28^ indicating that the GICs have achieved complete expansion. For EG and D-EG, a strong peak appears at 2*θ* around 26.3°, corresponding to the (002) plane of the graphite phase.^28^ After the introduction of diamond, the (002) peak was slightly shifted to the left, and the layer spacing was found to have increased from 0.334 nm to 0.336 nm by calculation. The crystallinity was decreased from 67.9 to 53.6. Narrow diffraction peaks are observed in D-EG powder at 2*θ* values near 44°, 75°, and 92°, corresponding to the (111), (220), and (311) facets of diamond.^13^

### Electrical conductivity and mechanical properties

Furthermore, an experimental investigation was conducted to assess the electrical conductivity and mechanical properties of different cathodes. It was established that higher conductivity facilitates charge efficiently transfer and helps to avoid scattering electrons.^29^ Furthermore, enhanced mechanical properties contribute to the preservation of the cathode’s structural integrity during the preparation process and the ability to withstand external mechanical shocks during extended use, thereby prolonging the service life.^1^ Figure 3C shows the electrical conductivity of the bulk cathode for different process parameters. It is evident that the electrical conductivity undergoes a significant decline with the incorporation of diamond. Furthermore, the conductivity gradually increases from 71 S/cm to 209 S/cm as the diamond content decreases, indicating that the diamond inside the composite cathode inhibits electron transport. Figure 3D shows the mechanical properties of the bulk composite cathode. The tensile strength of the bulk composite cathode gradually decreases with increasing diamond content from 5.99 MPa to 3.48 MPa. This is due to the fact that the presence of diamond makes the interior of the bulk composite cathode no longer compact, and it can easily fracture at these locations during tensile loading.^30^

### Field emission properties

**Figure 4:**
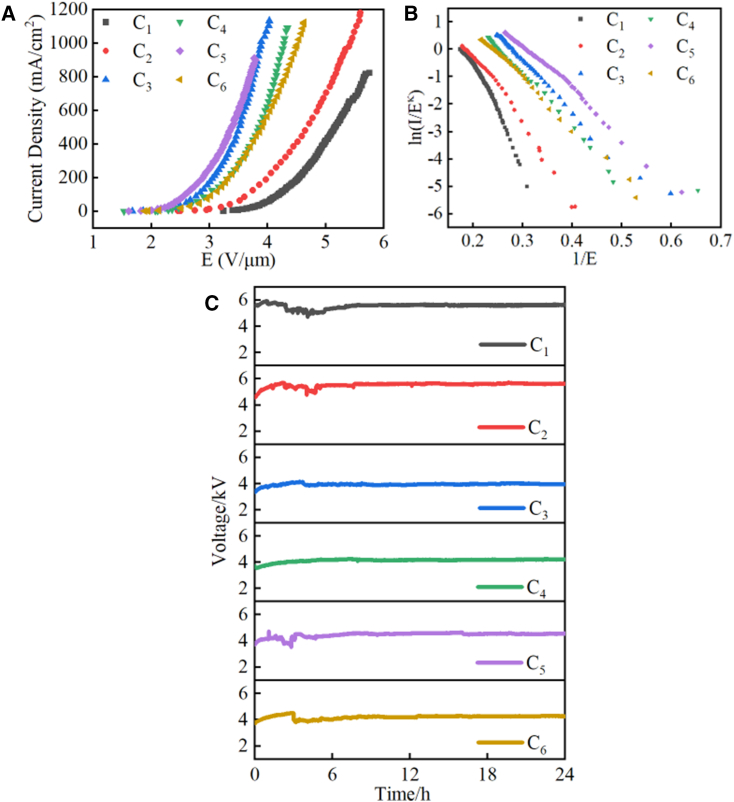
Field emission properties of composite cathodes (A–C) (A) *J-E* curves of composite cathodes C_1_ - C_6_, (B) *M-G* plots of C_1_ - C_6_, and (C) emission stability for cathodes C_1_ – C_6_ at a current of 4 mA.

Figure 4A shows the relationship curves between field emission current density (*J*) and electric field strength (*E*) for different types of bulk cathodes. The turn-on and threshold field strengths (*E*_*to*_ and *E*_*th*_) are defined as electric field strengths corresponding to 1 mA/cm^2^ and 10 mA/cm^2^, respectively. The calculated *E*_*to*_ and *E*_*th*_ are given in Table 2. To conduct a more detailed evaluation of the field emission performance of the composite cathode, the field emission relationship was properly evaluated using the Murphy-Good (MG) model (Figure 4B).^35^^,^^36^ The MG field emission equation gives the local emission current density (LECD) $J_{L}^{MG}$ in terms of the local work function *ϕ* and local barrier field *F*_*L*_:

(Equation 1) $$J_{L}^{MG}=t_{F}^{-2}J_{kL}^{SN}$$

(Equation 2) $$J_{kL}^{SN}=a\phi^{-1}F^{2}\;\exp\left[\frac{-v_{F}b\phi^{3/2}}{F_{L}}\right]$$

where *A* = 1.54 × 10^−6^AV^−2^eV and *B* = 6.83 × 10^3^eV^−3/2^Vμm^−1^, *v*_*F*_ is the value of *v*(*x*) that applies to the Schottky-Nordheim (SN) barrier defined by *ϕ* and *F*_*L*_, and *t*_*F*_ is the corresponding value of the special mathematical function (SMF) *t*(*x*) defined by:

(Equation 3) $$t\left(x\right)=v\left(x\right)-\left(\frac{4}{3}\right)x\frac{dv}{dx}$$

**Table 2:** Field emission properties of different types of cathodes

Cathode	*E*_*to*_(V/μm)	*E*_*th*_(V/μm)	Maximum current density (mA/cm^2^)	Field enhancement factor	Reference
C_1_	3.24	3.57	824.16 (at 5.75 V/μm)	2159	Varshney et al.^31^
C_2_	2.46	2.99	1182.45 (at 5.60 V/μm)	2862	Our work
C_3_	1.67	2.29	1134.94 (at 4.03 V/μm)	3977	
C_4_	1.53	2.36	1091.82 (at 4.34 V/μm)	4386	
C_5_	1.61	2.11	911.24 (at 3.77 V/μm)	4490	
C_6_	1.89	2.51	1123.85 (at 4.63 V/μm)	4057	
graphene-diamond hybrid films	2.4 (at 1 nA/cm^2^)	–	0.1 (at 15 V/μm)	1950	Santos et al.^32^
graphene-diamond hybrids	4.6 (at 1 μA/cm^2^)	–	3.2 × 10^−3^	2204	Li et al.^33^
diamond-graphite nanohybrid films	6.6 (at 11 μA/cm^2^)	–	1.6(at 9.9 V/μm)	1644	Wang et al.^34^

The MG plot has the form *ln*{*I*/*E*^*κ*^} vs. 1/*E*, where *κ* = 2−*η*/6 and *η* ≈ 9.836239(*eV*/*ϕ*)^1/2^. For *ϕ* = 5 eV, a typical value for the local work function of carbon-based materials,^37^ *κ* ≈ 1.27. From the MG plot, the effective field enhancement factors (FEFs) of the emitter were extracted by fitting straight lines to the data and using the relation *γ*_*eff*_ = −0.95*bϕ*^3/2^/*S*_*extr*_, where *S*_*extr*_ is the extracted slope. The FEF values are also listed in Table 2. As can be seen from Table 2, *E*_*to*_ and *E*_*th*_ of C_1_ were reduced from 3.24 V/μm and 3.57 V/μm to 1.53 V/μm and 2.11 V/μm, respectively, after mixing diamond. The maximum current density was increased from 824.16 mA/cm^2^ to 1182.45 mA/cm^2^. The *E*_*to*_ was reduced by more than half, indicating that the field emission performance of the composite cathode was significantly improved by mixing diamond. This is primarily due to the following factors: (i) The introduction of diamond has been shown to induce alterations in the emission surface, thereby enhancing the local electric field strength (Figures S3 and S4). This phenomenon is further evidenced by the alteration in the field enhancement factor (from 2159 to 4490). (ii) As demonstrated by the electron emission photographs (Figure S5), under conditions of low current, the EG emits electrons primarily from two sides, a phenomenon attributable to the local electric field enhancement effect. However, following the introduction of diamond, the edge effect is mitigated, resulting in the emission of a substantial number of electrons from the interior areas. (iii) The negative electron affinity potential property of diamond makes it easier for electrons to be emitted into the vacuum.^1^ Table 2 also lists other diamond composite cathodes, and it can be seen that the D-EG composite cathodes have better field emission properties.

In addition, when C_2_, C_3_, and C_4_ are compared, at a diamond size of 1.15 μm, *E*_*to*_ and *E*_*th*_ tend to decrease further as the diamond content decreases, indicating that more diamonds is not better. This is mainly due to the tendency to agglomerate as the number of diamonds increases, resulting in an electric field screening effect that inhibits electron emission (Figure S6). In addition, due to the extremely high hardness of diamond, the mechanical properties of the D-EG bulk composite cathode will further deteriorate as the diamond content increases, ultimately leading to difficulties in maintaining its own integrity after cold pressing. On the other hand, comparing C_3_, C_5_, and C_6_, for the same diamond content (20%), the composite cathode C_5_ with a diamond size of 0.5 μm has the lowest *E*_*to*_ and *E*_*th*_, indicating that C_5_ has the best electron emission performance. This is because 0.5 μm diamond has the highest local electric field strength. Reduced diamond size scatters electrons and affects electron transport, as evidenced by the lower conductivity of C_6_.

**Figure 5:**
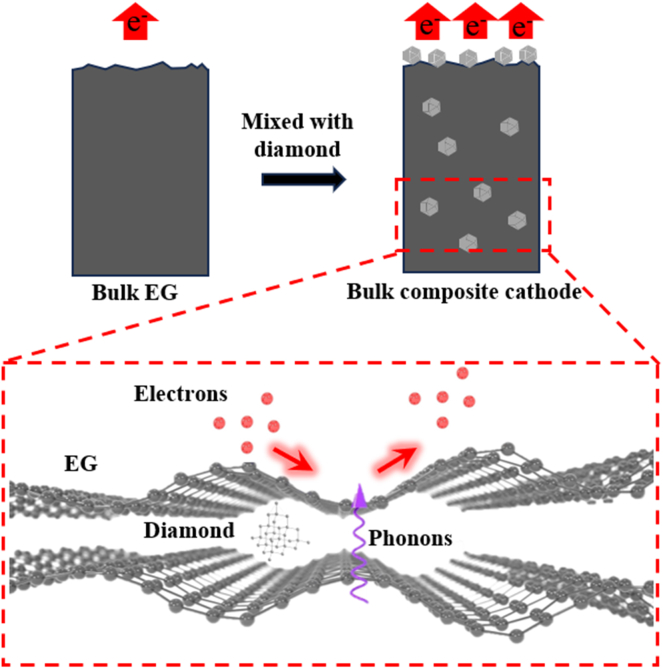
Schematic of electron emission and propagation

The principle of electron emission and propagation in the composite cathode can be explained in Figure 5. In the absence of diamond admixture, the predominant emission of electrons originates from the protrusion edge of the emitting surface of the bulk EG (Figure S5A). This configuration results in a reduced number of emission points, consequently leading to a diminished field emission performance. However, upon the introduction of diamond into the emitting surface, a transformation occurs wherein the diamond also participates in the emission as an emitting point (Figure S5B). This results in a substantial enhancement of the composite cathode field emission performance, which is attributable to the negative electron affinity potential characteristics inherent in the diamond.^1^ Moreover, graphite tends to lay flat on the emission surface, resulting in a reduction of the local electric field.^29^ Conversely, the introduction of diamond leads to the creation of a rougher emission surface (Figures S3 and S4) and an enhancement of the local electric field. Besides, within the composite cathode, phonons principally propagate along the EG, while diamond can provide a transmission path for phonons and improve the thermal conductivity of the composite cathode to achieve the rapid conduction of Joule heat.^1^^,^^2^

After 10 emission cycles, the stability of the field emission performance was assessed by constant current voltage variations, as shown in Figure 4C. Voltage values were recorded once per second at a constant current of 4 mA during the 24 h measurement period. For the majority of cathodes, a notable fluctuation in voltage was evident during the initial 6 h, subsequently exhibiting a stabilizing trend. This phenomenon can be attributed to the dislodgement of weakly bound particles from the cathode surface under the influence of ponderomotive forces. Additionally, the protrusions on the cathode surface are subjected to ion bombardment, resulting in their spraying.^24^ The fluctuation in voltage was calculated using the voltage at the commencement of the measurement period and the following equation^34^:

(Equation 4) $$\delta_{v}=\frac{\sum_{i=1}^{N}\left|V_{i}-V_{ave}\right|}{NV_{ave}}\times 100\%$$

where *V*_*i*_ is the voltage at each measurement point, and *V*_*ave*_ represents the average voltage. The calculated voltage fluctuations are 2.2%^38^ (C_1_), 1.8% (C_2_), 1.2% (C_3_), 1.8% (C_4_), 2.5% (C_5_), and 2.1% (C_6_). After mixing the diamond, only C_2_, C_3,_ and C_4_ show an increase in emission stability. This may be attributed to the existence of larger diamond particles within the cathode, which serve to enhance the overall resistance of the particles to the action of ponderomotive forces.^24^ Diamond’s high thermal conductivity, high chemical inertness, and high hardness inhibit self-heating/temperature rise, ion-induced erosion, and impurity-induced corrosion or oxidation, contributing to improved emission stability. In addition, the larger diamond particles provide greater coverage, protecting the underlying EG from external bombardment and effectively dissipating self-heating heat flow.^13^ However, as the diamond particles become smaller, the stability of C_5_ and C_6_ deteriorates instead. This indicates that the emission stability of the composite cathode is related to the size of the diamond particles. It could be explained by the fact that the desorption of particles from the cathode surface under the influence of the ponderomotive forces of the electric field affects the stability of the field emission.^24^

### Conclusions

In conclusion, this study investigated the field emission properties of bulk diamond/expanded graphite composite cathodes. The results of the field emission experiments demonstrate that the field emission performance of the D-EG composite cathode is significantly enhanced in comparison to the cathode without diamond and that emission stability is also improved. The composite cathode with a diamond particle size of 0.50 μm and a content of 20% has the best field emission performance, with *E*_*to*_ and *E*_*th*_ values of 1.61 V/μm and 2.11 V/μm, respectively. The maximum current density and field enhancement factor are 911.24 mA/cm^2^ (at 3.77 V/μm) and 4490, respectively.

### Limitations of the study

To facilitate more accurate comparisons, further research is necessary to ensure uniformity and consistency in the thickness of the block composite cathodes. Furthermore, processes such as hot-press sintering may be considered to achieve tighter bonding between different materials.

## Resource availability

### Lead contact

Further information and other requests should be directed to and will be fulfilled by the lead contact, Yihui Zhang (yhzhang0718@163.com).

### Materials availability

This work did not generate new unique reagents.

### Data and code availability

• All data reported in this article will be shared with the lead contact upon request.
• This article does not report the original code.
• Any additional information required to reanalyze the data reported in this article is available from the lead contact upon request.

## Acknowledgments

This work was supported by the Shaanxi Provincial Natural Fund Youth Project [2024JC-YBQN-0451], Shaanxi Province Postdoctoral Fund Program [2023BSHEDZZ54], and 10.13039/501100002858China Postdoctoral Science Foundation [2024M752565].

## Author contributions

Qianyu Ji: resources and writing – review and editing. Yihui Zhang: data curation and writing – original draft. Jiacheng Zhang: investigation. Wenhua Guo: conceptualization and resources. Jiyuan Zhao: resources and supervision.

## Declaration of interests

The authors declare no competing interests.

## STAR★Methods

### Key resources table

**Table:** 

REAGENT or RESOURCE	SOURCE	IDENTIFIER
**Chemicals, peptides, and recombinant proteins**		

Natural graphite flakes (32 mesh)	Qingdao Xinlei Graphite Products Co., Ltd	https://xinleiqd.cn.coovee.com/
Tetrabutylammonium perchlorate	MERYER Co., Ltd	CAS:1923-70-2
Propylene carbonate	MERYER Co., Ltd	CAS:108-32-7
Acetone	MERYER Co., Ltd	CAS:67-64-1
Absolute ethanol	MERYER Co., Ltd	CAS:64-17-5
N-methyl-2-pyrolidone	MERYER Co., Ltd	CAS:872-50-4
Diamond	Zhecheng Fangyuan Diamond Co., Ltd	https://shop72338471.taobao.com/

### Experimental modal and study participant details

This work did not include any experimental model or study participants.

### Method details

#### Fabrication of the bulk D-EG composite cathodes

The fabrication of the bulk D-EG composite cathodes can be divided into two steps. First step: D-EG powder was formatted in accordance with the route delineated in Figure 1. The synthesis of graphite intercalation compounds (GICs) was achieved through the utilization of the aforementioned methodology. A total of 400 mg of GICs and diamond granules (different mass fractions and different sizes) were placed in a mortar and manually ground for a period of 30 min in order to ensure thorough mixing. The ground mixture was placed in a crucible and subjected to microwave irradiation at 900 W for a period of 10 s, thereby obtaining the D-EG mixture.

Second step: the fabrication of the bulk D-EG composite cathodes was initiated through the process of cold pressing the grounded mixture (Figure S1). The D-EG mixture obtained in the previous step was homogeneously placed in a stainless steel mold, pressed at a pressure of 160 MPa for 10 min, and cut into long strips of D-EG bulk composite cathodes. The resulting cathodes were designated according to the nomenclature *C*_*n*_, where *n* represents the specific type of cathode. Cathode C_1_ was composed exclusively of EG. The cathodes C_2_-C_6_ were synthesized from a mixture of diamond granules and EG. All cathodes were cut into elongated strips with a height of 12 mm and a width of 3 mm. The thickness of these strips varied according to the diamond content and size. As illustrated in Table 1, the thickness of the cathodes C_1_-C_6_ is presented, in addition to the size and mass fraction of the diamond particles utilized.

#### Field emission measurement

Field emission performance was measured under vacuum (5 × 10^−4^ Pa) using a homemade diode device (Figure S2). The samples were tightly clamped in a fixture consisting of two stainless steel plates as the cathode, whereas a molybdenum sheet (radius 25 mm, thickness 1.5 mm) was employed as the anode. The gap between the anode and cathode was finely set as 1 mm. For ease of calculations, the emission area is defined as the cross-sectional area of the sample (i.e., the product of average thickness and width). Constant current mode is used for field emission performance testing. Each sample is tested 10 times continuously in the range of 0-10 mA, and the current-voltage curve obtained in the 10th test is used as the test data. After that, the emission stability was evaluated by observing the voltage fluctuation in constant current mode. All measurements were performed at room temperature.

#### Characterization

The morphology and microstructure of the samples were characterized by a field emission scanning electron microscope (MAIA3 LMH, Czech). Raman spectra were recorded by a laser Raman spectrometer (InVia Qontor, Hong Kong) using a 532 nm excitation laser. The laser power was 100 mW and the accumulation time was 10 s. X-ray diffraction (XRD) data was collected via a Bruker X-ray diffractometer (D8 ADVANC, Germany) with Cu Kα radiation. The electrical conductivity of the monoliths was measured by the four-wire (Kelvin) method (Keithley Model 2450, USA). The press was applied by a multifunctional static testing machine (UTM5305, SHENZHEN SUNS TECHNOLOGY STOCK CO., LTD.). The thickness of bulk field emitters was measured by a micrometer. Each sample was measured at 10 randomly selected points and averaged to obtain the thickness. The mechanical tests were performed using the Linkam MFS350 module.

### Quantification and statistical analysis

This article does not include statistical analysis.
