# Supplementary material for: Field electron emission properties of bulk diamond/expanded graphite composite cathode
Source: iScience. 2025 Nov 14;28(12):114043. doi: 10.1016/j.isci.2025.114043 (PMC12723154; doi:10.1016/j.isci.2025.114043)
Supplement: Document S1. Figures S1–S6 [file mmc1.pdf]

iScience, Volume *28*

Supplemental information

Field electron emission properties of bulk diamond/expanded graphite composite cathode

Qianyu Ji, Yihui Zhang, Jiacheng Zhang, Wenhua Guo, and Jiyuan Zhao

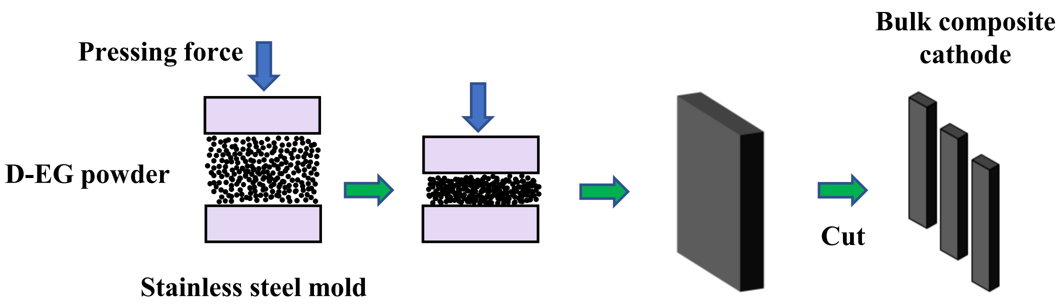

Figure S1. A schematic representation of the fabrication process of the bulk field emitters.

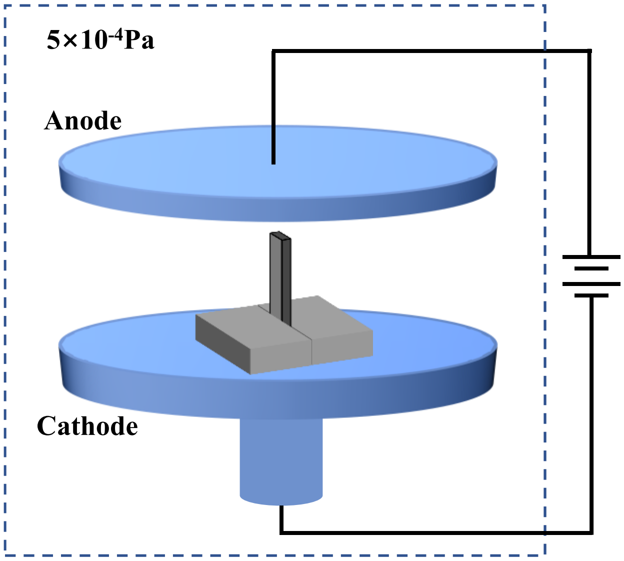

Figure S2. Schematic of the bulk emitters field emission measurements.

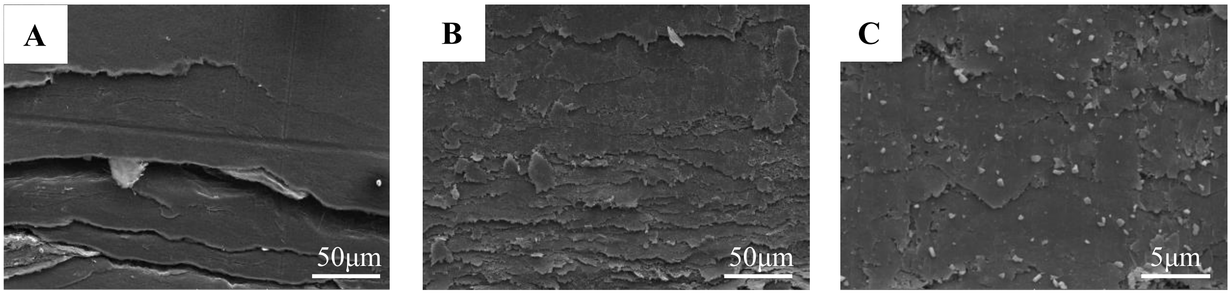

Figure S3. SEM images of C_1 and C_5 emission surface before field emission: (a) C_1; (b) C_5; (c) high magnification of C_5.

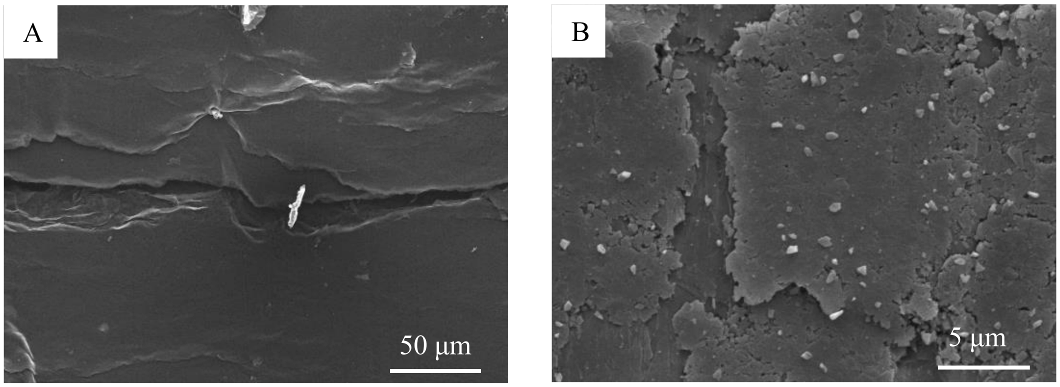

Figure S4. SEM images of C_1 and C_5 emission surface after field emission: (a) (a) C_1; (b) C_5.

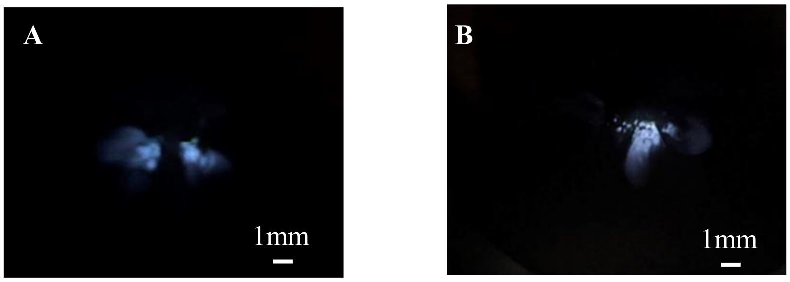

Figure S5. Optical photographs of the electron distributions of (a) C_1, (b) C_5 at current 0.4mA.

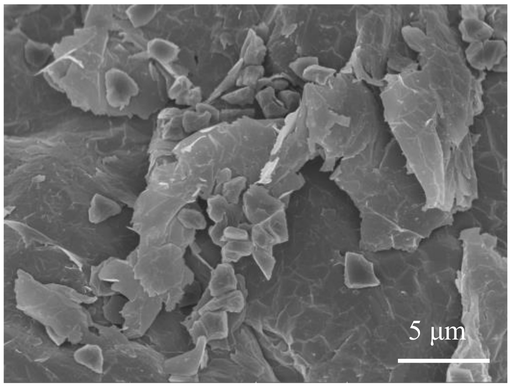

Figure S6. SEM image of C_2.
